# Oxidative Stress in Muscle Diseases: Current and Future Therapy

**DOI:** 10.1155/2018/6439138

**Published:** 2018-04-26

**Authors:** Andrey Jorge Serra, Marko D. Prokić, Andrea Vasconsuelo, José Renato Pinto

**Affiliations:** ^1^Universidade Nove de Julho (UNINOVE), São Paulo, SP, Brazil; ^2^University of Belgrade, Belgrade, Serbia; ^3^Universidad Nacional del Sur, Bahia Blanca, Argentina; ^4^Department of Biomedical Sciences, College of Medicine, Florida State University, Tallahassee, FL, USA

Oxidative stress can be considered a consequence of imbalance in the formation of reactive oxygen species (ROS) and antioxidant defense systems, in which mitochondria appear to be the main source of ROS production. A key concern is that high levels of ROS can modulate molecular and structural modifications and lead to functional changes within the muscle. In this regard, ROS causes oxidation of biomolecules, in which results in loss of their biological functions and leads to homeostatic imbalance. The main effects mediated by ROS relate to its potential to cause oxidative damage in cells and tissues [1]. Therefore, a ROS burst contributes to cellular dysfunction and can cause a wide range of chronic diseases. Scientific advances have allowed a better understanding of the role of oxidative stress on muscular homeostasis; thereby, deciphering and understanding how ROS regulate specific molecules and processes that alter physiological function could cause development of pathologies. Thus, studying the effects of ROS could assist with the development of new therapeutic avenues that address a wide range of muscle disorders.

This special issue encompasses cutting-edge research and review articles that focus on the role of oxidative stress in both acute and chronic progressive muscle disorders. This special issue provides a platform for sharing recent scientific advancements with researchers and practitioners who work in both skeletal and cardiac muscle scopes, which include clinical diagnostics, basic scientists, and physicians interested in physiology and physiopathology of muscle. Thus, articles included in this special issue address the molecular and cellular mechanisms involved in these processes as well as current therapeutic approaches in human and animals.

Two investigations in this special issue focus on physiological changes induced by acute exercise and subsequent oxidative stress in male runners who completed a marathon race in two different thermal conditions—hot and temperate environments, as well as a rat model of resistance exercise. In these studies, marathon running performance decreased up to 10%, while the wet bulb globe temperature increased from 10 to 25°C [2]. The investigation also highlighted that hot temperature induces pronounced homeostatic alterations, including a proinflammatory response, limited muscle oxygenation, and increased oxidative stress [3, 4]. In the study by H. A. de Oliveira et al., male marathon runners were studied to determine the effects of environmental temperature variation on hematological profiles; tissue damage and oxidative stress markers were investigated in men. The performance of marathon runners was reduced 13.5% in a hot environment (31.4°C) compared when they were performing at a lower temperature (19.8°C). In both environments, marathon racing promotes fluid and electrolyte imbalances, hemolysis, immune activation, tissue damage, and oxidative stress. Moreover, long-term exercise during heat stress conditions worsened running performance by causing hematological changes due to fluid and electrolyte imbalances and purine/protein oxidation of erythrocytes, which lead to renal damage and immune activation. On the other hand, the authors have concluded that reduced performance at hot environment is not associated with muscle damage and lipid peroxidation.

Exercise-induced increases in free radical formation are thought to play a key role in the mechanisms by which skeletal muscle adapts to exercise training [5, 6], whereas aerobic physical exercise appears to be the main trigger of free radical formation. In addition, resistance exercise can also evoke increased free radical production in muscle because of the occurrence of intermittent bouts of hypoxia/reperfusion. An excessive increase in oxidative stress or a free radical burst in working muscles can override the physiological benefits of exercise causing extensive damage to proteins and cellular dysfunction [7, 8]. This is a key concern during intense training stages, in which the burst in free radicals can overcome the existing antioxidant mechanisms. In fact, studies have reported beneficial effects of antioxidant agents used to counteract the increase in oxidative stress induced by exercise. In this special issue, R. A. Oliveira et al. applied low-intensity laser therapy in rat gastrocnemius muscle prior to a high-intensity resistance exercise session. The laser application brought about improvements in lipoperoxidation levels and the oxidized protein content in muscle. Moreover, laser therapy appeared to increase activity of antioxidant enzymes (i.e., superoxide dismutase and glutathione peroxidase) and led to higher total nonenzymatic antioxidant potential. Thus, photobiomodulation could be a promising antioxidant approach to counter risks associated with high-intensity resistance exercises. However, further studies are needed to better understand the influence of different irradiation dosages and the mechanisms that modulate oxidative stress linked to exercise.

Further analysis of the role of photobiomodulation was showed in a review article entitled “Effects of Photobiomodulation Therapy on Oxidative Stress in Muscle Injury Animal Models: A Systematic Review,” which moves the focus on from the several models of experimental muscle injury (e.g., fatigue, cryoinjury, and traumatic insult). First, S. A. dos Santos et al. summarized that, despite the limited number of studies in this research area, photobiomodulation could be a factual approach to reduce oxidative stress markers (e.g., thiobarbituric acid reactive) and to increase antioxidant substances (e.g., superoxide dismutase, catalase, and glutathione peroxidase). Second, the authors' advocate “whole mechanisms” by which photobiomodulation could modulate oxidative stress generated during muscle injury. A possible mechanism is the improvement of mitochondrial function, which is associated with reduced free radical formation linked to superoxide dismutase, catalase, and glutathione peroxidase. The primary beneficial effect of photobiomodulation in different muscles may be its anti-inflammatory effects [9, 10]. In this regard, S. A. dos Santos et al. have considered that oxidative muscular homeostasis linked to photobiomodulation may be mediated by its anti-inflammatory action, which mitigates the migration of inflammatory cells (e.g., neutrophils and macrophages) and thus the source of ROS.

This special issue also covers the role of oxidative stress and antioxidants in different disorders using human cells and model organisms. A. J. O'Leary et al. explored the cellular and functional consequences of sustained hypoxic stress in a mouse model of acute hypoxia and the effects of N-acetyl cysteine (NAC) pretreatment. The authors showed that acute hypoxic stress increases the protein content of HIF-1*α* in the diaphragm as well as activated MAPK, mTOR, Akt, and FoxO3a signaling pathways. Acute hypoxic stress also led to increases in lipid peroxidation and FoxO3 and MuRF-56 1 gene expression in the diaphragm. The treatment with NAC at a dose of 200 mg/kg completely prevented hypoxia-induced diaphragm dysfunction in mouse models studied. NAC substantially enhanced cell survival signaling pathways which, along with its general antioxidant functions, prevented hypoxia-induced diaphragm oxidative stress. These findings may have relevance to develop treatment strategies for hypoxaemic respiratory patients and populations living at high altitudes. NAC treatment was also evaluated in a study conducted by A. Michelucci et al., in which RYR1^Y522S/WT^ knock-in mouse, carrying a human mutation in RYR1 linked to malignant hyperthermia (MH) exhibiting *cores* with a NAC treatment regimen for either two or six months. They found that two months of NAC-treatment initiated at two months of age, when mitochondrial and fiber damage was still minimal, (i) reduced the formation of *unstructured* and *contracture cores*, (ii) improved muscle function, (iii) and decreased mitochondrial damage. The beneficial effects of NAC treatment was also evident following six months of treatment that was initiated later, at four months of age when structural damage was advanced. Likely, NAC exerts its protective effects by lowering oxidative stress, as supported by the reduction of 3-NT and Mn-superoxide dismutase levels. This work suggests that NAC-administration is beneficial and prevents mitochondrial damage and the formation of *cores* and improves muscle function in RYR1^Y522S/WT^ mice. S. C. Khor et al. evaluated the effects of a 24 h treatment with the antioxidant tocotrienol-rich fraction (TRF), with the goal of reestablishing the oxidative status of myoblasts during replicative senescence. The effects of TRF on oxidative status were compared to the effects of other antioxidants (*α*-tocopherol (ATF) and NAC). Treatment with TRF was associated with diminished ROS production and lipid peroxidation in senescent myoblasts. Moreover, gene expression in senescent myoblasts of Mn-superoxide dismutase, catalase, and glutathione peroxidase was modulated by TRF. Additional parameters were affected by TRF including increased activity of superoxide dismutase and catalase and reduced glutathione peroxidase in senescent myoblasts. In comparison to ATF and NAC, TRF was more efficient at increasing antioxidant capacity and reducing insult caused by free radicals. The authors suggested that TRF can ameliorate antioxidant mechanisms and improve replicative senescence-associated oxidative stress in myoblasts.

An imbalance between oxidant and antioxidant systems has been proposed as a secondary effect in Duchenne muscular dystrophy (DMD, X-linked genetic disease). In this regard, the significance and precise extent of the perturbation in redox signaling cascades are poorly understood. L. Pelosi et al. tried to fill this gap of knowledge. The authors reported that mdx dystrophic mice can trigger a compensatory antioxidant response at the presymptomatic stage of the disease. On the other hand, increased circulating levels of IL-6 perturb the redox signaling cascade, even prior to the development of necrosis, which is responsible for the severity and progressive nature of DMD disease. Multiple cellular and molecular mechanisms have been implicated in the DMD, which makes the disease complex to study and complicates the development of beneficial interventions. In this regard, M. D'Agostino et al. showed that miR-200c overexpression impaired skeletal muscle differentiation in cultured myoblasts and anti-miR-200c treatment ameliorated myogenic differentiation. Moreover, the authors found that miR-200c and p66Shc Ser-36 phosphorylation was increased in mdx muscles. These findings link miR-200c to muscle wasting in DMD, in which miR-200c overexpression can be evoked by ROS.

Accumulation of reactive oxygen species (ROS) can be mitigated by inbuilt mechanisms that help prevent excessive oxidative stress in cardiomyocytes during hypoxia-reoxygenation. Nitric oxide (NO) can compete successfully with oxygen when the myocardium experiences hypoxia. Therefore, NO accumulation can serve in a protective role by forming NO-Fe^2+^ complexes when chelating iron is released from oxygen complexes [11]. The study by V. I. Kapelko et al. explored the use of a stable NO donor (Oxacom®) (DNIC) dinitrosyle iron complex with glutathione to preserve the integrity of cardiomyocytes and improve functional outcomes in rat hearts after hypoxia-reoxygenation. This study is the first to report antihypoxic cardioprotective potential of Oxacom to counter hypoxic insult, including a reduction in hypoxic contractures, attenuation of cardiac arrhythmias, and an increase of left ventricular pressures during reoxygenation. Oxacom pretreatment substantially reduced ROS accumulation in hypoxia-treated rat cardiomyocytes. The reduction in ROS is expected to improve the performance of ion transporters and regulatory proteins, maintaining their thiol groups in a reduced state and protected by reversible S-nitrosylation [12, 13]. Oxacom eliminates slow sarcoplasmic Ca^2+^ removal as well as delayed relaxation of cardiomyocytes. These findings dovetail well, with the overall view that primary Ca^2+^ removal from the sarcoplasm by the energy-dependent ion transporters/exchangers is slowed down in hypoxia since high-energy phosphates and oxygen are depleted. Some of the protective effects of Oxacom may be derived by its impact on improving mitochondrial function. This will benefit contractile processes overall, which have even greater energy requirements. Overall, the authors conclude that Oxacom positively affects energy production and utilization, which may underlie its ability to prevent hypoxic contractures and the rapid functional recovery seen during reoxygenation.

Mechanisms to protect against oxidative stress appear to diverge between the different sexes. A study by A. Michelucci et al. addresses the unexplained sex differences that underlie vulnerability for developing malignant hyperthermia (MH), which is influenced by oxidative status. MH is a lethal disorder in humans, triggered by halogenated/volatile anesthetics, and caused by the release of excessive calcium in skeletal muscle [14, 15]. Estrogens protect calsequestrin-1 (CASQ1) knockout mice during lethal hyperthermic episodes, and the mechanism of protection relies on a reduction of oxidative stress in muscle. When male CASQ1-null mice are exposed to conditions that resemble human MH (halothane and heat), they experience a high incidence of mortality [16]. To explore the role of sex hormones in development of MH, A. Michelucci et al. treated male and female CASQ1-null mice with Premarin (conjugated estrogens) and Leuprolide (GnRH analog), respectively, for one month. Their findings demonstrate that previously vulnerable CASQ1-null males were largely protected from MH-induced mortality by Premarin treatment prior to exposure to halothane and heat, while CASQ1 females treated with Leuprolide suffered higher mortality rates after losing the protective effects of estrogen. To explore functional changes in these mice due to the hormonal treatments, individual muscles were exposed to temperature and caffeine. The sarcoplasmic Ca^2+^ release was measured in single fibers and levels of oxidative stress, and expression of the main redox regulators was measured. In summary, Premarin treatment in CASQ1-null males reduced muscle temperature and caffeine sensitivity, normalized the release of Ca^2+^ from the sarcoplasmic reticulum (SR), and reduced oxidative stress. Overall, female mice appear to be protected by female sex hormones from lethal hyperthermic episodes by reducing the amount of sarcoplasmic reticulum Ca^2+^ leak and oxidative stress.

An epidemiological study by E. Brunelli et al. examined a healthy population in Italy consisting of woman and man volunteers to look for early indicators of major cardiovascular risk factors [17–19]. Blood plasma from individuals was analyzed for prooxidant and antioxidant molecules. In high cardiovascular risk categories, a significant depletion in total plasma antioxidant barrier efficacy was found. As expected, increasing age correlated with increased oxidative status. In addition, individuals with metabolic disorders including diabetes, obesity, and dyslipidemia displayed significant depletion of the efficacy of total plasma antioxidant barrier. Known risk factors, such as cholesterol imbalance, appear to be the main element leading to depletion of total plasma-antioxidant barrier efficacy. Furthermore, minimal increases in both total serum cholesterol and total serum cholesterol/HDL as well as hypertension and slight increases in systolic blood pressure significantly increase oxidative status. In summary, this study identifies a primary prevention target and depletion of the antioxidant barrier, as the earliest detectable redox disturbance. This redox perturbation appears to pinpoint the likelihood that an individual will develop cardiovascular disease in the future due to overutilization of antioxidant-directed mechanisms.

We hope that this special issue is successful in providing new insights into the impact of oxidative stress in muscle on different physiological and pathological disorders. The findings presented here can be used to improve knowledge and understanding of changes induced by exercise on oxidative stress, in which low-intensity laser therapy is able to modulate excessive oxidative stress after physical effort. We also feel that this is an opportunity to report findings concerning the role of N-acetyl cysteine in different *in vitro* and *in vivo* models. Moreover, the editors considered the interdisciplinary nature of the papers included as necessary to expand on concepts fundamental to basic research as well as those important to applied science. As a result, papers covering the effects of oxidative stress in Duchenne muscular dystrophy, cardiomyocyte hypoxia-reoxygenation, malignant hyperthermia, and cardiovascular risk were included. Therefore, the readers of this special issue should find information of interest, relevant to their respective area of scientific investigations.

## References

[1] Halliwell B., Whiteman M. (2004). Measuring reactive species and oxidative damage *in vivo* and in cell culture: how should you do it and what do the results mean?. *British Journal of Pharmacology*.

[2] Ely M. R., Martin D. E., Cheuvront S. N., Montain S. J. (2008). Effect of ambient temperature on marathon pacing is dependent on runner ability. *Medicine & Science in Sports & Exercise*.

[3] Cheuvront S. N., Kenefick R. W., Montain S. J., Sawka M. N. (2010). Mechanisms of aerobic performance impairment with heat stress and dehydration. *Journal of Applied Physiology*.

[4] Gill S. K., Teixeira A., Rama L. (2015). Circulatory endotoxin concentration and cytokine profile in response to exertional-heat stress during a multi-stage ultra-marathon competition. *Exercise Immunology Review*.

[5] Bailey D. M., Lawrenson L., McEneny J. (2007). Electron paramagnetic spectroscopic evidence of exercise-induced free radical accumulation in human skeletal muscle. *Free Radical Research*.

[6] Gomez-Cabrera M. C., Domenech E., Romagnoli M. (2008). Oral administration of vitamin C decreases muscle mitochondrial biogenesis and hampers training-induced adaptations in endurance performance. *The American Journal of Clinical Nutrition*.

[7] Retamoso L. T., Silveira M. E. P., Lima F. D. (2016). Increased xanthine oxidase-related ROS production and TRPV1 synthesis preceding DOMS post-eccentric exercise in rats. *Life Sciences*.

[8] Wiecek M., Maciejczyk M., Szymura J., Szygula Z. (2017). Effect of maximal-intensity exercise on systemic nitro-oxidative stress in men and women. *Redox Report*.

[9] Ferraresi C., Huang Y. Y., Hamblin M. R. (2016). Photobiomodulation in human muscle tissue: an advantage in sports performance?. *Journal of Biophotonics*.

[10] Manchini M. T., Serra A. J., Feliciano R. . S. (2014). Amelioration of cardiac function and activation of anti-inflammatory vasoactive peptides expression in the rat myocardium by low level laser therapy. *PLoS One*.

[11] Korge P., Ping P., Weiss J. N. (2008). Reactive oxygen species production in energized cardiac mitochondria during hypoxia/reoxygenation: modulation by nitric oxide. *Circulation Research*.

[12] Gyorke S., Terentyev D. (2008). Modulation of ryanodine receptor by luminal calcium and accessory proteins in health and cardiac disease. *Cardiovascular Research*.

[13] Figueiredo-Freitas C., Dulce R. A., Foster M. W. (2015). *S*-Nitrosylation of sarcomeric proteins depresses myofilament Ca^2+^ sensitivity in intact cardiomyocytes. *Antioxidants & Redox Signaling*.

[14] MacLennan D., Phillips M. (1992). Malignant hyperthermia. *Science*.

[15] Rosenberg H., Sambuughin N., Riazi S., Dirksen R., Adam M. P., Ardinger H. H., Pagon R. A. (2003). Malignant hyperthermia susceptibility. *GeneReviews*.

[16] Dainese M., Quarta M., Lyfenko A. D. (2009). Anesthetic- and heat-induced sudden death in calsequestrin-1-knockout mice. *The FASEB Journal*.

[17] Kannel W. B., Dawber T. R., Kagan A., Revotskie N., Stokes J. (1961). Factors of risk in the development of coronary heart disease—six year follow-up experience: the Framingham study. *Annals of Internal Medicine*.

[18] Levy D., Wilson P. W. F., Anderson K. M., Castelli W. P. (1990). Stratifying the patient at risk from coronary disease: new insights from the Framingham Heart Study. *American Heart Journal*.

[19] Hobbs F. D. R. (2004). Cardiovascular disease: different strategies for primary and secondary prevention?. *Heart*.

